# An update on ICES’ Applied Health Research Question (AHRQ) program: Informing public health policy and practice using linked, population-based administrative health data in a collaborative model.

**DOI:** 10.23889/ijpds.v9i5.2855

**Published:** 2024-09-18

**Authors:** Diana An, Minnie Ho, J. Michael Paterson, Refik Saskin, Luke Mondor, Clare Atzema, Lesley Plumptre, Jeruby Retnakanthan, Dina Skvirsky, Charles Victor, Susan E. Bronskill

**Affiliations:** 1 ICES

## Objective

To provide an update on the key characteristics, refinements to project flow, and future opportunities for a program providing customized research evidence to health system policymakers and providers.

## Approach

With a goal to inform health system decision making, the program answers research questions posed by health system requestors that can be answered with linked, population-based administrative data. Now in its 10th year of operation, the program has been refined over time to improve efficiency, usefulness of research products, and requestor satisfaction. Satisfaction surveys and individual-level engagements with requestors are used to collect feedback on the needs of an increasingly diverse group of requests.

## Results

With 508 requests, 82 Data Sharing Agreements, and 257 unique requestors since its inception, the program informs governmental decision making, evaluates intervention effectiveness, and aids grassroots organizations’ planning for services. Notable updates to the program include, inserting multiple opportunities for connection between the program and requestors through the project life cycle to understand goals and needs; assigning staff scientists to shepherd projects and promote efficiency; training coordinators to review privacy impact assessments; and (soon) accepting a broader range of projects to include data beyond the health sector e.g. education.

## Conclusion

LiThis program continues to improve and is an exemplar of sustainable approaches to supporting health system requestors with evidence from administrative data in program evaluation, planning and policy change.

## Implications

Being responsive to requestor needs has allowed the program to attract an increasingly diverse range of requesters who can obtain impactful and timely research evidence.

